# Momordicine-I, a Bitter Melon Bioactive Metabolite, Displays Anti-Tumor Activity in Head and Neck Cancer Involving c-Met and Downstream Signaling

**DOI:** 10.3390/cancers13061432

**Published:** 2021-03-21

**Authors:** Subhayan Sur, Robert Steele, T. Scott Isbell, Kalyan Nagulapalli Venkata, Mostafa E. Rateb, Ratna B. Ray

**Affiliations:** 1 Department of Pathology, Saint Louis University, St. Louis, MO 63104, USA; subhayan.sur@health.slu.edu (S.S.); Robert.steele@health.slu.edu (R.S.); scott.isbell@health.slu.edu (T.S.I.); 2 Department of Pharmaceutical and Administrative Sciences, Saint Louis College of Pharmacy, St. Louis, MO 63104, USA; Kalyan.Venkata@uhsp.edu; 3 School of Computing, Engineering & Physical Sciences, University of the West of Scotland, Paisley PA1 2BE, Scotland, UK; Mostafa.Rateb@uws.ac.uk; 4 Cancer Center, Saint Louis University, St. Louis, MO 63104, USA

**Keywords:** momordicine-I, bitter melon (*Momordica charantia*), head and neck cancer, C-MET signaling, therapy

## Abstract

**Simple Summary:**

The incidence of head and neck cancer (HNC), one of the most aggressive cancers, is increasing rapidly globally. Conventional and targeted therapies show limited success with several undesirable side effects. Thus, there is a critical clinical need to identify additional alternative therapeutic strategies for successfully managing the disease. Preclinical and clinical studies indicate the crucial roles of dietary phytochemicals to manage different cancers. We and others previously showed the potential anticancer effect of bitter melon extract (BME) to prevent various cancers, including HNC. In this study, we identified momordicine-I (M-I) as a bioactive component in the BME. Subsequent mechanistic study showed that M-I inhibited HNC cell (JHU022, JHU029, Cal27) proliferation involving c-Met and downstream signaling. In pre-clinical mouse models, M-I showed similar effectiveness to prevent HNC tumor growth in mice with no apparent toxic side effect, suggesting an additional option for HNC therapy.

**Abstract:**

Head and neck cancer (HNC) is one of the most aggressive cancers, and treatments are quite challenging due to the difficulty in early diagnosis, lack of effective chemotherapeutic drugs, adverse side effects and therapy resistance. We identified momordicine-I (M-I), a bioactive secondary metabolite in bitter melon (*Momordica charantia*), by performing liquid chromatography-high resolution electrospray ionization mass spectrometry (LC-HRESIMS) analysis. M-I inhibited human HNC cell (JHU022, JHU029, Cal27) viability in a dose-dependent manner without an apparent toxic effect on normal oral keratinocytes. Mechanistic studies showed that M-I inhibited c-Met and its downstream signaling molecules c-Myc, survivin, and cyclin D1 through the inactivation of STAT3 in HNC cells. We further observed that M-I was non-toxic and stable in mouse (male C57Bl/6) blood, and a favorable pharmacokinetics profile was observed after IP administration. M-I treatment reduced HNC xenograft tumor growth in nude mice and inhibited c-Met and downstream signaling. Thus, M-I has potential therapeutic implications against HNC.

## 1. Introduction

Head and neck cancer (HNC) arises from the mucosal surfaces of the oral cavity, oropharynx, larynx, paranasal sinuses, nasal cavity, and salivary glands, and is considered the sixth most common cancer worldwide [1]. Major risk factors for HNC are habitual tobacco and alcohol consumption, chewing betel quid, chewing tobacco and human papillomavirus (HPV) infection. In 2021, the estimated incidence rate of cancers in the oral cavity and pharynx is 54,010, with an associated 10,850 deaths in the USA [2]. Despite the advancements in surgical techniques, chemotherapy and radiation therapy, treatment of the disease is very challenging for both clinicians and patients. The overall survival rates are 40–50%, which have not improved over the past few decades. Difficulty in performing early diagnosis, lack of early detection markers, adverse side effects, lack of effective chemotherapeutic drugs, therapy resistance and economic expense of conventional therapies make the disease management difficult. The epidermal growth factor receptor (EGFR) inhibitors are the only approved drugs for targeted therapy with limited success and resistance [3,4]. Compensatory activation of another receptor kinase mesenchymal-epithelial transition factor (c-Met) potentially reduces the efficacy of anti-EGFR inhibitors [3]. Aberrant activation of c-Met signaling is frequently observed in HNC and is associated with poor prognosis and metastasis [3,4,5]. The c-Met signaling shares common down-stream targets with EGFR and induces HNC cell proliferation, migration, invasion, and metastasis. Several agents that target c-Met have been developed, and results appear promising in HNC preclinical studies. Clinical studies using c-Met inhibitors and monoclonal antibodies are in developing stages. Programmed cell death receptor (PD-1) monoclonal antibodies nivolumab and pembrolizumab were approved in 2016 to treat the advanced and therapy resistance cases [3,4]. However, PD-1 monotherapy generates adaptive resistance and takes a longer time to achieve clinical response than other conventional therapies [6]. Thus, the development of additional therapeutic strategies is necessary for successfully managing the disease.

Phytochemicals and their derivatives from plant or microbial sources are promising alternative therapeutic option. While conventional therapy shows limited success due to resistance and adverse side effects, several natural products have showed promising results in preclinical studies [7,8,9]. Some phytochemicals have been reported to be able to target multiple molecules in signaling pathways for the control of cancer cell growth and cancer prevention, while being inexpensive and devoid of toxic effects [8,9]. Many clinical trials using bioactive secondary metabolites are completed with promising outcomes and some are ongoing. Many drugs derived from plant or microbial sources like vinca alkaloids (vinblastine, vincristine, vindesine, vinorelbine), taxanes (paclitaxel, docetaxel), podophyllotoxin and its derivations (topothecan, irinothecan), anthracyclines (doxorubicin, daunorubicin, epirubicin, idarubicin) are already approved [9,10]. 

Our group and others have evaluated the potential anticancer effect of bitter melon *(Momordica charantia)* extract (BME) in several cancers [11,12,13,14,15,16]. In HNC preclinical models, BME prevents HNC cell proliferation targeting c-Met and downstream signaling, inhibits glucose and lipid metabolism, induces cell death, and enhances the immune defense system [17,18,19,20,21]. Bitter melon belongs to the family Cucurbitaceae, and is cultivated in tropical and sub-tropical regions of Asia, Africa, and South America. The plant has the highest nutritional values among other cucurbits. It contains diverse secondary metabolite classes, including cucurbitane type triterpenes, phenolic acids, flavonoids, essential oils, sterols, saponin, and primary metabolites, including fatty acids, amino acids, lectins, and some proteins [11,12,16]. Among the isolated compounds, momordica antiviral protein, 30 kD (MAP30), α momorcharin, RNase MC2, kuguacin J, α eleostearic acids and lectins showed anticancer effect in different models [16]. However, limited follow-up studies were reported with the compounds in preclinical models. This study aimed to identify the bioactive metabolite(s) from the BME and evaluate its role in HNC preclinical mouse model. Our results highlighted momordicine-I (M-I) as a potent active component in the BME. M-I is non-toxic, stable in blood and acts similarly to the extract as HNC growth inhibitor involving c-Met and downstream signaling in both in vitro and in vivo models. This is the first study describing the therapeutic potential of M-I for the regression of HNC tumors in a pre-clinical model. 

## 2. Results

### 2.1. Identification of Active Ingredients in Bitter Melon Extract (BME)

**Figure 1:**
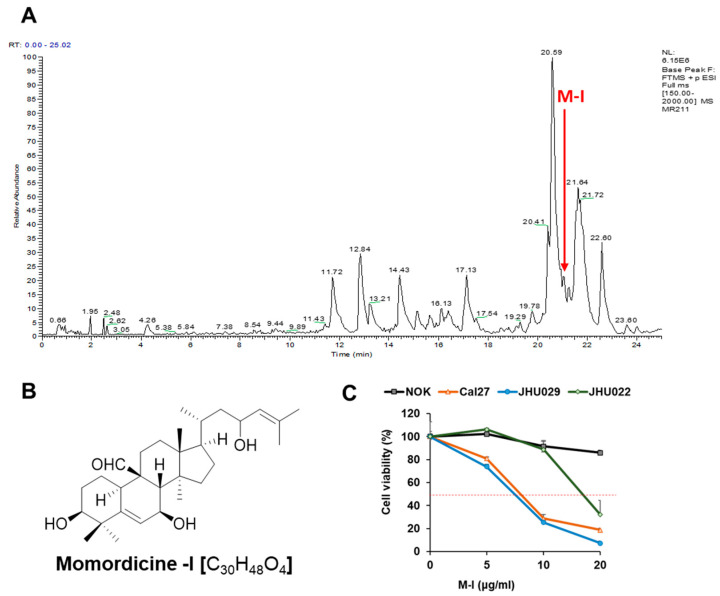
Mass-spectrometric analysis of bitter melon extract and identification of momordicine-I. (**A**) LC trace of bitter melon extract done by HRMS. The red arrow indicated the presence of momordicine-I (M-I) peak. (**B**) Chemical structure of momordicine-I. (**C**) HNC cells (Cal27, JHU029, JHU022) and control NOK were treated with M-I at different concentration for 48 h, and cytotoxicity assay was performed. Small bar indicates standard error.

We and others have reported the potential anticancer effect of BME in several cancer models [11,12,13,14,15,16]. The biological activity of BME depends on its chemical constituents. Among several ingredients in bitter melon (like phenolic acids, flavonoids, essential oils, fatty acids, amino acids, lectins, sterols, saponin and proteins), cucurbitane type triterpenoids and cucurbitane type triterpene glycosides are a major chemical class in the family Cucurbitaceae and are suggested to be responsible for bitterness and much of the biological activities in the family [16]. We performed liquid chromatography coupled to high resolution electrospray ionization mass spectrometry (LC-HRESIMS) analysis to identify the cucurbitane type triterpenoids and triterpene glycosides present in the BME. The LC-HRESIMS data revealed the presence of a total of 28 secondary metabolites, of which 4 metabolites belonged to cucurbitane type triterpenoids and 20 belonged to cucurbitane triterpene glycosides in the extract (Table 1, Figure 1A). The BME also contained a cucurbitane triterpenoid at a retention time 21.64 min with a molecular formula of C_33_H_48_O_7_ that was not reported previously. The molecular formula and MS/MS analysis indicated this compound to be a new derivative of 7,23-dihydroxy-3-*O*-malonylcucurbita-5,24-dien-19-al with an extra double bond. Additionally, the HRMS analysis indicated the presence of three monoterpenoid glycosides and one oleanane-type triterpene saponin. It worth noting that many of those metabolites have unknown biological functions, otherwise reported in Table 1.

### 2.2. M-I Inhibits HNC Growth In Vitro

Due to their limited reported biology, we have selected a few triterpenoids and triterpene glycosides, based on their availability, for preliminary in vitro screening against HNC (JHU022, JHU029, Cal27) cell lines. We identified momordicine-I (M-I) as one of the chemical constituents at the retention time of 21.06 min in the BME (Figure 1A). The M-I [C_30_H_48_O_4_, PubChem CID: 14807332; IUPAC Name: (3*S*, 7*S*, 8*S*, 9*R*, 10*R*, 13*R*, 14*S*, 17*R*) 3,7-dihydroxy-17-(4-hydroxy-6-methylhept-5-en-2-yl)-4,4,13,14-tetramethyl-2,3,7,8,10,11, 12,15,16,17-decahydro-1*H*-cyclopenta[a]phenanthrene-9-carbaldehyde] is a white crystalline solid that belongs to the cucurbitane-type triterpene class (Figure 1B). We performed cytotoxicity assays of M-I in HNC cells Cal27, JHU029 and JHU022 and observed that M-I inhibited HNS cells in a dose-dependent manner (Figure 1C). However, M-I had a minimum effect on human normal oral keratinocytes (NOK). The IC_50_ doses of Cal27, JHU022 and JHU029 cells treated with M-I for 48 h were 7 µg/mL, 17 µg/mL and 6.5 µg/mL, respectively. Other compounds, momordicoside K (MK), and karavilagenin D (KD), were also examined on HNC cell cytotoxicity assay. These, two other compounds required much higher amounts (>50 µg/mL) even for ~40% cell death (Figure S1). 

### 2.3. M-I Inhibits c-Met Signaling in HNC Cells

**Figure 2:**
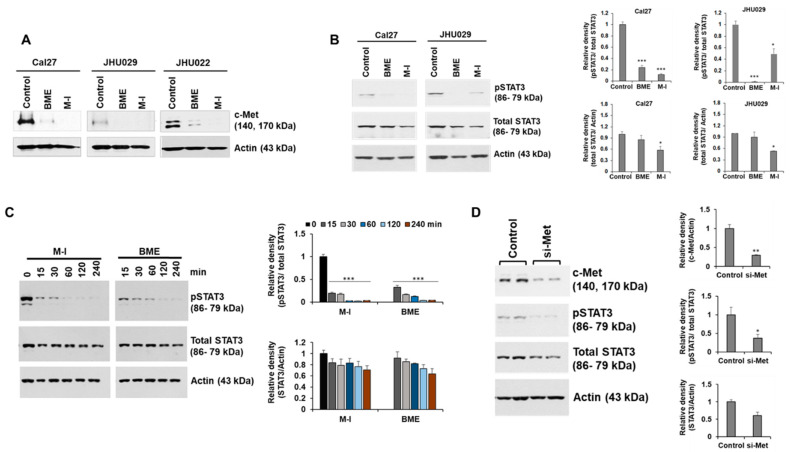
Momordicine-I inhibited c-Met signaling in HNC cells. (**A**) Cal27, JHU029 and JHU022 cells were treated with either 2% BME or 10 µg/mL of M-I and JHU022 cells were treated with either 3% BME or 20 µg/mL dose of M-I. Cell lysates were prepared after 48 hr of treatment and subjected to Western blot analysis using specific antibodies. Representative Western blot images for c-Met expression in Cal27, JHU029 and JHU022 cells are shown. Membrane was reprobed with the antibody for actin as an internal control. (**B**) Representative Western blot image for phospho-STAT3 (pSTAT3 Tyr-705) and total STAT3 expression in Cal27 and JHU029 cells with BME or M-I treatment. Membrane was reprobed by actin as an internal control. Quantitative representation of Western blot band intensities (right panel). Small bar indicates standard error (* *p* < 0.05; *** *p* < 0.001). (**C**) Cal27 cells were treated with 2% BME or M-I (10 μg/mL) at indicated time points. (**D**) Cal27 cells were treated with control siRNA or siRNA to c-Met (si-Met) for 48 hr. Cell lysates (from panels c and D) were subjected to analyze by western blot for pSTAT3 or STAT3 using specific antibody. Membranes were reprobed by actin as internal control. Quantitative representation of Western blot band intensities (right panel)**.** Small bar indicates standard error (*** *p* < 0.001). Uncropped western blots figures are shown in Figures S2–S4.

**Figure 3:**
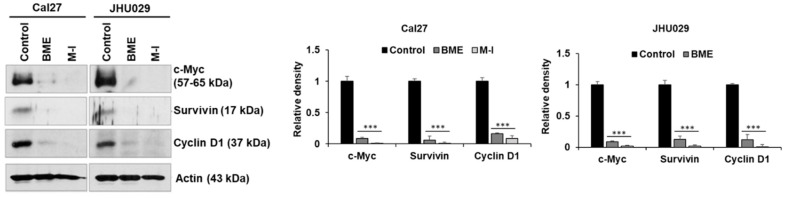
Momordicine-I inhibits downstream of c-Met signaling in HNC cells. Representative Western blot image for c-Myc, survivin and cyclin D1 expression in Cal27 and JHU029 cells with treatment of BME or M-I. The same membrane used in panel A was reprobed. Actin was used as internal control. Quantitative representation of Western blot band intensities (right panel). Small bar indicates standard error (*** *p* < 0.001). Uncropped western blots figures are shown in Figure S5.

**Figure 4:**
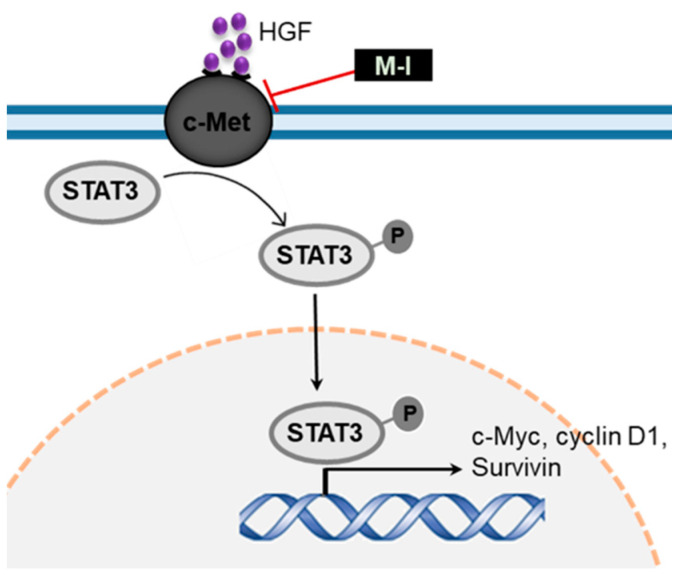
Schematic representation showing mode of action of M-I in inhibition of c-Met signaling. Sharp arrows indicate activation/ induction and blunt arrows indicate inhibition.

We observed that BME inhibited c-Met signaling and its downstream signaling molecules to prevent HNC growth [17]. To investigate the mechanistic effect of M-1, Cal27, JHU029 and JHU022 cells were treated with M-I for 48 hr along with vehicle control. BME was used in parallel as a control. A significant reduction of c-Met expression was observed in these cells following BME or M-I treatment (Figure 2A). The receptor tyrosine kinase c-Met induces tumor development by activating multiple downstream molecules, including the oncogenic transcription factor signal transducer and activator of transcription 3 (STAT3) [22,23]. Upon activation of c-Met signaling, STAT3 is activated by phosphorylation at the Tyr-705 residue, dimerized and translocated to the nucleus for activation of several proliferation and survival related genes including c-Myc, survivin and cyclin D1 [23,24]. Activated STAT3 can also transcribe STAT3 gene as a positive feedback mechanism [25]. We observed a significant reduction in phospho-STAT3 (Tyr-705) expression following treatment with BME or M-I in Cal27 and JHU029 cells (Figure 2B). A substantial reduction in total STAT3 level was also noted in M-I treated cells. Next, we examined whether the short term (15, 30, 60, 120, 240 min) BME or M-I treatment can inhibit p-STAT3 levels to ascertain whether initially M-1 inhibits p-STAT3 levels without affecting total STAT protein levels. Interestingly, we have observed that BME or M-I treatment on HNC cells inhibits pSTAT3 in 15 min without affecting total STAT3, however, after 240 min of treatment, total STAT3 was inhibited (Figure 2C). This may be due to the feedback mechanism of pSTAT3 on STAT3 regulation as discussed earlier [25]. Further, depletion of c-Met by specific siRNA inhibits pSTAT3 (Figure 2D). We also observed a significant inhibition of STAT3 downstream molecules; c-Myc, survivin and cyclin D1, in Cal27 and JHU029 cells following treatment with BME and M-I (Figure 3). Taken together, our results suggested that M1 inhibits c-Met signaling in the prevention of HNC growth (Figure 4).

### 2.4. Pharmacokinetic and Toxicity Profile of M-I

**Figure 5:**
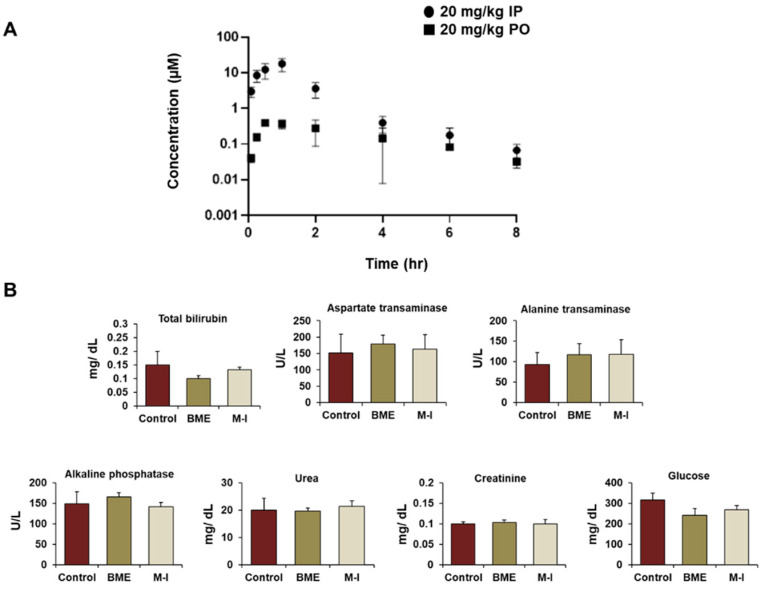
In vivo pharmacokinetic and toxicity profile of momordicine-I. (**A**) Concentration-time profile of momordicine-I in C57Bl/6 male mice at 20 mg/kg single dose administered by IP injection or oral gavage (PO). Data is from *n* = 3 mice and represents mean ± standard deviation. (**B**) Comparison of metabolic panel from serum among M-I (30 mg/kg/mouse) and BME treated groups with untreated control mice. Small bar indicates standard error.

To evaluate the pharmacokinetic (PK) profile of M-I, a single dose (20 mg/kg) was given to C57Bl/6 male mice by either intraperitoneal injection (IP group; *n* = 3) or oral ga-vage (oral group; *n* = 3). A dose of 20 mg/kg was chosen based on previous publications [26,27]. The average plasma concentration ± standard deviation at each time point is shown in Figure 5A and corresponding PK parameters are summarized in Table 2. M-I was rapidly absorbed with a maximum plasma concentration 1 h post-IP and PO dose. Cmax values were 18 µM and 0.5 µM after the single 20 mg/kg IP and PO dose, respec-tively. The observed elimination half-life was 0.9 h in the IP group and 2 h in the PO group. The oral group had loose stool, starting four hours post-dose, which had not re-solved by 8 h but all mice appeared normal after twenty-four hours. No adverse events were observed for the mice in the IP dosing group. We therefore selected IP administration of M-I for subsequence studies.

For toxicity analysis, mice (*n* = 3) were given 20 mg/ kg of M-I twice a day or 30 mg/kg IP dose of M-I once a day by IP injection for five days. In parallel, there were untreated control (*n* = 3) and BME treated group (*n* = 3) for comparison. Behavior and body weight of mice were monitored daily. The body weight in all the mice was stable, with no drastic changes seen in any of the mice tested. We collected blood on day six, and serum chemistries related to liver and kidney function were examined. The concentration of total bilirubin, alanine transaminase, aspartate transaminase, alkaline phosphatase, creatinine, urea, and glucose were comparable in M-I and BME treated groups with untreated control mice, indicating no toxic effect (Figure 5B).

### 2.5. Therapeutic Potential of M-I in HNC Xenograft Model 

**Figure 6:**
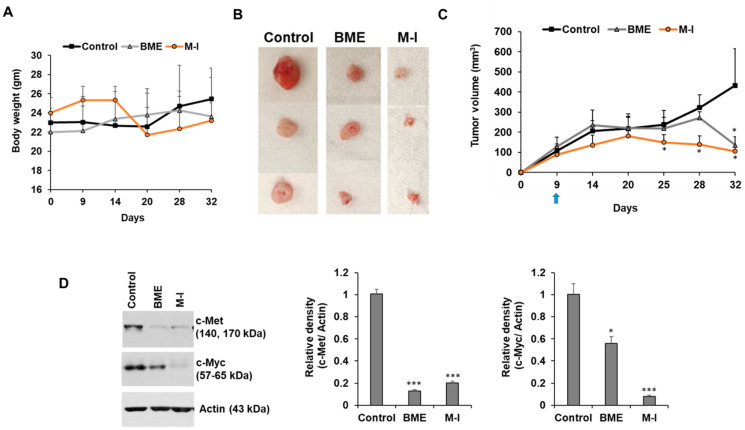
Therapeutic effect of momordicine-I in JHU029 xenograft model. JHU029 cells (1.5 × 10^6^) cells were injected subcutaneously into the flank of nude mice. After the formation of a palpable tumor, mice were randomly divided into three groups (*n* = 5): Control (without any treatment), BME group (30% BME through drinking water) and M-I group (30 mg/kg IP, once in a day and every day). (**A**) Body weight was measured in control and treated mice. (**B**) Representative images of tumors in control and treatment groups. (**C**) Tumors were measured using a slide caliper and tumor volumes were calculated. Arrow indicates starting point BME/M-I treatment. (**D**) Control or treated tumor lysates were subjected to Western blot analysis for c-Met and c-Myc expression using specific antibodies and representative bands are shown. The blot was reprobed with an antibody to Actin for normalization. Right panel shows quantitation. Small bar indicates standard error (*, *p* < 0.05; *** *p* < 0.001). Uncropped western blots figures are shown in Figure S6.

We investigated the therapeutic efficacy of M-I in HNC xenograft model. For this, we implanted JHU029 cells in flanks of nude mice. After the formation of a palpable tumor, mice were divided into three groups: untreated control (*n* = 5), BME treated group (*n* = 5), and M-I treated group (*n* = 5). In the BME group, mice were given 30% BME through drinking water as described previously [20]. The mice in M-I group received 30 mg/kg of M-I through IP, once a day till the end of the experiment. Body weight and tumor volume were measured. We observed a sudden drop in body weight (~10%) in three out of five mice in M-I group on Day 20; however, they gained back the weight (Figure 6A). We also observed the formation of fluid in the tumors as reported previously [17,28]. Some of the tumors in the control group had an open wound; therefore we needed to sacrifice all the animals on day 32. The BME and M-I group mice displayed significantly reduced tumor volume (Figure 6B,C). However, M-I showed a better effect in reducing tumor growth. We examined c-Met signaling in control and treated tumors. We observed a significant reduction in expression of c-Met and its downstream molecule c-Myc in BME and M-I treated groups compared to untreated control tumors (Figure 6D). 

**Figure 7:**
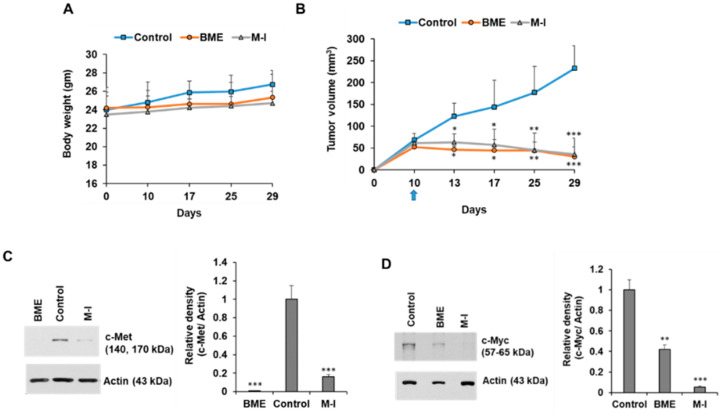
Therapeutic effect of momordicine-I in Cal27 xenograft model. Cal27 cells (1.5 × 10^6^) cells were injected subcutaneously into the flank of nude mice. After formation of palpable tumor, mice were randomly divided into three groups (*n* = 5): Control (without any treatment), BME group (30% BME through drinking water) and M-I group (30 mg/kg IP, once in a day and every day). (**A**) Body weight was measured in control and treated mice. (**B**) Tumors were measured using a slide caliper and tumor volumes were calculated. Arrow indicates starting point BME/ M-I treatment. (**C**,**D**) Control or treated tumor lysates were subjected to Western blot analysis for c-Met and c-Myc expression using specific antibodies and representative bands are shown. The blot was reprobed with an antibody to Actin for normalization. Right panel shows quantitation. Small bar indicates standard error (*, *p* < 0.05; **, *p* < 0.01; *** *p* < 0.001). Uncropped western blots figures are shown in Figure S7.

For further validation, we also examined the effect of M-I in Cal27 xenograft model. We observed increasing body weight in all the mice throughout the experiment, and ~50% reduction of tumor growth in BME or M-I treated xenograft tumor (Figure 7A,B). We further observed a significant reduction in c-Met and c-Myc expression in BME and M-I treated groups compared to the control (Figure 7C,D). Our results demonstrated that M-I has the potential as a therapeutic candidate for HNC treatment. 

## 3. Discussion

In this study, we identified M-I as a bioactive secondary metabolite using LC-HRESIMS analysis of the BME. We observed that M-I inhibits c-Met signaling in HNC cell (JHU022, JHU029, Cal27) lines. We further demonstrated a significant regression of tumor growth in HNC xenograft models following daily administration of M-I with no toxicity, suggesting the therapeutic efficacy of BME and M-I by targeting c-Met signaling. 

Bitter melon plant and its fruit contain many phytochemicals, and among those cucurbitane type triterpenoids and cucurbitane type triterpene glycosides are the major chemical constituents [16]. The cucurbitane-type triterpenoids and cucurbitane-type triterpene glycosides are suggested to be responsible for bitterness and confer much of the biological activities of the plant [16,29]. M-I belongs to the class cucurbitane-type triterpene. This secondary metabolite was first identified and characterized in leaves and vines of *Momordica charantia* L. [30]. In our study, we identified M-I in the water extract of the fruit. Although the compound was identified in 1984, the biological function of M-I was not well evaluated. M-I was recently reported to have inhibitory effects on high-glucose-induced cell proliferation and collagen synthesis in rat cardiac fibroblasts [31] and stimulate insulin secretion in vitro [32], but to the best of our knowledge, the anticancer effect of M-I was not reported. We initially screened a few metabolites from bitter melon and got a significant inhibition with M-I with IC_50_ of less than 8 μg/mL in Cal27 and JHU029 cells. We observed that M-I inhibited HNC growth and c-Met signaling. Aberrant activation of c-Met signaling through overexpression of c-Met and its downstream molecules c-Myc, cyclin D1, and survivin were observed predominantly in HNC [3,4,5], which were reduced following M-I treatment. Increased c-Met signaling is associated with HNC progression and metastasis, and c-Met signalling inhibition by neutralizing antibody inhibited tumor growth and its metastatic potential [3,5].M-I may have other targets to inhibit cell proliferation which was not investigated in this study. We observed a significant reduction of tumor growth in two HNC xenograft models following BME treatment with no toxicity. Further, treatment of M-I (30 mg/kg/mouse) once a day worked similarly to twice a day in our pilot experiment (20 mg/kg/mouse). 

HNC therapy often has limited success. Resistance to approved anti-EGFR therapy sometimes makes the treatment management difficult [3,5]. Thus, targeting c-Met is suggested to be a promising alternative strategy. Many drugs that target c-Met signaling have been developed, showing promising results in preclinical and clinical studies. However, these drugs manifested several adverse side effects [3,5,33]. c-Met inhibitor GEN-203 and compound **8** showed liver and bone marrow toxicity in mice and myocardial degeneration in rats. Foretinib caused fatigue, hypertension, and gastrointestinal toxicities. Golvatinib caused supraventricular tachycardia, convulsion, and pulmonary embolism. Tivantinib (ARQ197) showed adverse events, including leukopenia, anemia, and neutropenia in clinical trial. Thus, identifying a new natural and non-toxic c-Met signaling inhibitor would have a high impact on HNC treatment, and M-I might be one of the major contributors with biological activity in BME. However, the functional mechanism of M-I mediated c-Met signaling inhibition remains to be further elucidated. Effect on M-I in the presence of an intact immune system would need further investigation in future. 

## 4. Materials and Methods

### 4.1. Cell Culture, Preparation of Bitter Melon Extract (BME) and Momordicine I (M-I)

Normal oral keratinocytes (NOK) (kindly gifted by Dr. Karl Mugner, Tuffs University, Boston, MA, USA) were maintained in Keratinocyte SFM medium supplemented with EGF and bovine pituitary extract (GIBCO, Life Technologies, Berkeley, MO, USA) and 1% penicillin/ streptomycin. HNC cell line Cal27 was purchased from the ATCC. JHU029 (JHU-29) and JHU022 (JHU-22) cell lines were procured from the Johns Hopkins University (Baltimore, MD, USA). The Cal27 and JHU022 cells were maintained in Dulbecco’s Modified Eagle Medium (DMEM), and JHU029 cells were in RPMI1640 (Sigma, St. Louis, MO, USA) media supplemented with 10% FBS and 1% penicillin/ streptomycin (Sigma-Aldrich, St. Louis, MO, USA) in a humidified CO_2_ incubator. The cell lines are routinely tested in our laboratory to rule out mycoplasma contamination using a commercial MycoAlert™ Mycoplasma Detection kit (Lonza, Morrisville, NC, USA). Cal27 cells are tongue origin and JHU022 and JHU029 cells are from laryngx origin. 

Bitter melon extract (BME) was prepared from the Chinese variety of young bitter melons (raw and green) as described previously [17]. Briefly, BME was prepared by aqueous extraction from whole fruit without seeds using a household juicer at room temperature with subsequent centrifugation at 15000× g at 4 °C for 30 min. BME was stored at −80 °C for further analysis. Cal27 and JHU029 cells were treated with 2% BME and JHU022 cells were treated with 3% BME as described previously [17,21]. The momordicine-I (>98% pure) was purchased from Chemfaces (Cat. No.: CFN92076; Hubei, China). The powder was dissolved in DMSO and added to the cells at different concentrations. Based on cytotoxicity data, Cal27 and JHU029 cells were treated with 10 µg/mL and JHU022 cells were treated with 20 µg/mL dose of M-I and incubated for 48 hr for further experiments. All the experiments were done at least in triplicate. 

### 4.2. Cytotoxicity Assay

Cal27, JHU029, JHU022 and NOK were seeded in 96 well-plate (5000 cells/well) and cells were treated with different concentration of M-I for 48 h. There were untreated control and DMSO treated vehicle control group for comparison. Cytotoxicity assay was performed using Cell Counting Kit-8 (Dojindo Molecular Technology, Rockville, MD, USA) according to manufacturer instruction.

### 4.3. Protein Isolation and Western Blot Analysis

Lysates from control, BME or M-I treated cells and tumors were prepared using 2× SDS sample buffer and subjected to western blot analysis using specific antibodies to c-Met (1: 500, Cell Signaling Technology, CST, Denvers, MA, USA,), phospho- STAT3 (pSTAT3-Tyr-705) (1:1000, CST), total STAT3 (1:1000, CST), c-Myc (1:1000, CST), survivin (1:500, Santa Cruz Biotechnology, SBT, Dallas, TX, USA) and cyclin D1 (1: 500, SBT). HRP conjugated anti-mouse or anti-rabbit secondary antibodies were purchased from Bio-Rad (Hercules, CA, USA). The blot was reprobed with HRP conjugated β-actin antibody (1:5000; SBT) to compare protein load in each lane. Densitometry analysis was done using Image J software (NIH, Bethesda, MD, USA).

### 4.4. Liquid Chromatography High Resolution Electrospray Ionization Mass Spectrometry (LC-HRESIMS)

HRESIMS analysis of the BME was done using a LTQ Orbitrap spectrometer coupled to an HPLC system (PDA detector, PDA autosampler, and pump, ThermoFisher Scientific, Inchinnan, Renfrew PA4 9R, UK). The following conditions were used: capillary voltage of 45 V, capillary temperature of 260 °C, auxiliary gas flow rate of 10−20 arbitrary units, sheath gas flow rate of 40−50 arbitrary units, spray voltage of 4.5 kV, and mass range of 100−2000 amu (maximal resolution of 30,000). For LC-HRESIMS, a Sunfire C_18_ analytical HPLC column (5 μm, 4.6 mm × 150 mm) was used with a mobile phase of 0 to 100% MeOH over 20 min followed by 100% MeOH over 5 min at a flow rate of 1 mL min^−1^. 

### 4.5. Pharmacokinetic and Toxicity Study

The pharmacokinetic (PK) study of M-I was performed in male C57Bl/6 mice. A sin-gle dose of M-I (formulated at 2 mg/mL dissolved in 5% DMSO/95% of a 30% *w*:*v* Captisol solution) was administered either by intraperitoneal injection (IP group, *n* = 3) or oral ga-vage (PO group, *n* = 3). After dosing, 20 µL blood samples were collected into heparin pre-coated tubes at 5 min, 15 min, 30 min, 1 h, 2 h, 4 h, 6 h, 8 h and 24 h. Samples were centrifuged, and the plasma collected. M-I concentration was determined using liq-uid chromatography-mass spectrometry/mass spectrometry (LC-MS/MS) as described earlier [34]. 

For toxicity study, C57Bl/6 male mice were received either 30% BME through drinking water or 20 mg/kg of M-I twice a day or 30 mg/kg of M-I once a day by IP injection for 5 days. There were three mice in each group, including untreated control. Blood was collected on day 6, and serum was prepared. Serum parameters related to hepatotoxicity (total bilirubin, alanine transaminase, aspartate transaminase, and alkaline phosphatase) and nephrotoxicity (urea and creatinine) were performed. 

### 4.6. Tumorigenicity Assay

JHU029 or Cal27 cells (1.5 × 10^6^) containing 40% Matrigel were injected subcutaneously into the flank of BALB/c athymic nude mice (7–8 weeks old). When the palpable tumor was developed (>60 mm^3^), mice were divided into three groups randomly, 5 mice in each group. The control group was without any treatment. The BME group received 30% (v/v) BME through drinking water. M-I group received 30 mg/kg dose of M-I once in day, every day. Body weight was monitored, and tumor size was measured using a slide caliper and volume was calculated using the formula ½ L × W^2^. After animal sacrifice tumors were dissected out and snap frozen in liquid nitrogen for further analysis. All the animal experiments were carried out in accordance NIH guidelines, following a protocol approved (1017) by the Institutional Animal Care and Use Committee (IACUC) of Saint Louis University.

### 4.7. Statistical Analysis

The results are presented as means ± standard deviations. Data were analyzed by Student’s *t*-test. *p* value of <0.05 was considered statistically significant. All experiments were repeated at least three times except animal experiments, and representative data are shown.

## 5. Conclusions

In conclusion, this is the first report showing M-I, a secondary metabolite from bitter melon, inhibited tumor growth in HNC xenograft models with no apparent toxicity. Mechanistic data demonstrated that M-I impairs c-Met signaling in HNC (JHU022, JHU029, Cal27) cells, which is schematically illustrated in Figure 4. Thus, M-I could be used as an additional chemotherapeutic agent or its structural motif to be developed against HNC.

## Figures and Tables

**Table 1:** LC-HRESIMS analysis of bitter melon extract.

Retention Time	Accurate Mass *m/z*	Suggested Formula ^a^	Tentative Identification ^b^	Reported Biology	Chemical Class
11.72	949.53672	C_47_H_80_O_19_	Momordicoside B	---	Cucurbitane triterpenoid glycosides
11.95	969.50531	C_49_H_76_O_19_	Goyasaponin III	---	Oleanane-type triterpene saponin
12.84	819.47360	C_41_H_70_O_16_	Momorcharaside A	---	Cucurbitane triterpenoid glycosides
13.21	813.46320	C_42_H_68_O_15_	Momordicoside O	---	Cucurbitane triterpenoid glycosides
14.43	797.46797	C_42_H_68_O_14_	Karaviloside X	---	Cucurbitane triterpenoid glycosides
14.53	797.46814	C_42_H_68_O_14_	Momordicoside N	---	Cucurbitane triterpenoid glycosides
14.65	801.49957	C_42_H_72_O_14_	Momordicoside C	---	Cucurbitane triterpenoid glycosides
15.13	781.47327	C_42_H_68_O_13_	Goyaglycoside F	---	Cucurbitane triterpenoid glycosides
15.63	657.42089	C_35_H_60_O_11_	Momorcharaside B	---	Cucurbitane triterpenoid glycosides
16.13	387.20139	C_19_H_30_O_8_	Vomifoliol β-D-glucopyranoside	---	Monoterpenoid glycosides
16.41	447.22241	C_21_H_34_O_10_	Sacranoside A	nitric oxide inhibitory effect	Monoterpenoid glycosides
17.13	653.42599	C_36_H_60_O_10_	Karaviloside XI	Antidiabetic	Cucurbitane triterpenoid glycosides
17.54	635.41531	C_36_H_58_O_9_	Momordicoside L	Weak α-glucosidase inhibition	Cucurbitane triterpenoid glycosides
19.78	649.43109	C_37_H_60_O_9_	Momordicoside K	---	Cucurbitane triterpenoid glycosides
20.41	649.43114	C_37_H_60_O_9_	Goyaglycoside A	---	Cucurbitane triterpenoid glycosides
20.59	663.44660	C_38_H_62_O_9_	Goyaglycoside C	---	Cucurbitane triterpenoid glycosides
20.65	315.18017	C_16_H_26_O_6_	Myrtenyl O-β-D-glucopyranoside	---	Monoterpenoid glycosides
20.74	615.38922	C_36_H_54_O_8_	Charantoside VII	---	Cucurbitane triterpenoid glycosides
20.80	619.42041	C_36_H_58_O_8_	Momordicoside V	---	Cucurbitane triterpenoid glycosides
20.95	631.42040	C_37_H_58_O_8_	Charantoside I	---	Cucurbitane triterpenoid glycosides
21.05	633.43618	C_37_H_60_O_8_	Charantoside V	---	Cucurbitane triterpenoid glycosides
21.06	473.3627	C_30_H_48_O_4_	Momordicine I	stimulate insulin secretion in vitro diabetes-associated cardiac fibrosis.	Cucurbitane-type triterpene
21.28	635. 4518	C_37_H_62_O_8_	Karaviloside III	cytotoxic activity against Hep3B and HepG2 cell lines	Cucurbitane triterpenoid glycosides
21.54	649.46745	C_38_H_64_O_8_	Karaviloside II	---	Cucurbitane triterpenoid glycosides
21.64	557.34720	C_33_H_48_O_7_	No hit-new malonylcucurbita-trien-19-al derivative	---	Cucurbitane-type triterpene
21.72	559.36299	C_33_H_50_O_7_	7,23-Dihydroxy-3-O-malonylcucurbita-5,24-dien-19-al	---	Cucurbitane-type triterpene
21.91	601.40955	C_36_H_56_O_7_	Charantoside IV		Cucurbitane triterpenoid glycosides
22.60	437.34125	C_30_H_44_O_2_	(23*E*)-Cucurbita-5,23,25-triene-3,7-dione	---	Cucurbitane-type triterpene

^a^ High-Resolution Electrospray Ionization Mass Spectrometry (HRESIMS) using XCalibur 3.0 and allowing for M + H/M + Na adduct. The suggested formulae are based on the Quasimolecular [M + H]^+^ form; ^b^ The suggested compounds were identified according to Dictionary of Natural Products (DNP 23.1, 2015 on DVD) and Reaxys online database.

**Table 2:** Pharmacokinetics parameters of momordicine I (M-I) administered either through intraperitoneally (IP) or orally (PO) in C57Bl/6 male mice (data provided as mean ± SD).

Pharmacokinetic Parameters	C57Bl/6 Male	
	20 mg/Kg-IP	20 mg/Kg-PO
T_½_ (h)	0.90 ± 0.02	2.11 ± 0.3
T_max_ (h)	1.00 ± 0	1.00 ± 0.9
C_max_ (ng/mL)	8427 ± 3419.4	214 ± 18.6
C_max_ (µM)	17.83 ± 7.2	0.45 ± 0.04
AUC_last_ (min*ng/mL)	762,559 ± 319,312.5	38,584 ± 16,628.4
AUC_INF_obs_ (min*ng/mL)	765,026 ± 320,184.6	41,251 ± 17,124.75
AUC (%Extrap)	0.33 ± 0.08	6.84 ± 1.5

Abbreviations: T½: Elimination half-life; Tmax: time to reach maximum (peak) plasma concentration following drug administration; Cmax: maximum (peak) plasma drug concentration; AUClast: area under the plasma concentration-time curve from time zero to time of last measurable concentration; AUCINF_obs: area under the concentration-time curve extrapolated from zero up to infinity; AUC (%Extrap): area under the first moment of the plasma concentration-time curve extrapolated from time t to infinity as a percentage of total AUC; Cl_obs: apparent total body clearance of the drug from plasma.

## Data Availability

The data generated for this study is included in this manuscript.

## Supplementary Materials

The following are available online at https://www.mdpi.com/2072-6694/13/6/1432/s1, Figure S1: caption, Figure S2: Whole blot for Figure 2A, Figure S3: Whole blot for Figure 2B, Figure S4: Whole blot for Figure 2C,D, Figure S5. Whole blot for Figure 3, Figure S6: Whole blot for Figure 6D, Figure S7: Whole blot for Figure 7C,D.

## Abbreviation

HNC	head and neck cancer
BME	bitter melon extract
M-I	momordicine-I
IUPAC	international union of pure and applied chemistry
MAP30	Momordica antiviral protein, 30 kD
NOK	normal oral keratinocyte
IC_50_	half maximal inhibitory concentration
c-Met	mesenchymal-epithelial transition factor
c-Myc	Myc proto-oncogene protein
STAT3	signal transducer and activator of transcription 3
EGFR	epidermal growth factor receptor
PD-1	programmed cell death protein 1
LC-HRESIMS	liquid chromatography coupled to high resolution electrospray ionization mass spectrometry
PK	pharmacokinetics
T½	elimination half-life
Tmax	time to reach maximum (peak) plasma concentration following drug administration
Cmax	maximum (peak) plasma drug concentration
AUClast	area under the plasma concentration-time curve from time zero to time of last measurable concentration
AUCINF_obs	area under the concentration-time curve extrapolated from zero up to infinity
AUC (%Extrap)	area under the first moment of the plasma concentration-time curve extrapolated from time t to infinity as a percentage of total AUC
Cl_obs	apparent total body clearance of the drug from plasma
