# Supplementary material for: Momordicine-I, a Bitter Melon Bioactive Metabolite, Displays Anti-Tumor Activity in Head and Neck Cancer Involving c-Met and Downstream Signaling
Source: Cancers (Basel). 2021 Mar 21;13(6):1432. doi: 10.3390/cancers13061432 (PMC8003975; doi:10.3390/cancers13061432)
Supplement: Supplementary file 1 [file cancers-13-01432-s001.pdf]

Supplementary Materials: Momordicine-I, a Bitter Melon Bioactive Metabolite, Displays Anti-Tumor Activity in Head and Neck Cancer Involving c-Met and Downstream Signaling

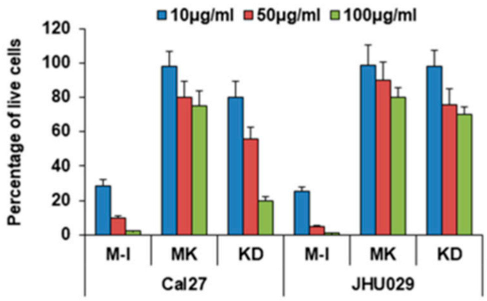

Figure S1. Cal27 and JHU029 ells were treated with momordicine-I (M-I), momordicosideK (MK), karavilageninD (KD) at indicated doses for 48 hr and cytotoxicity assay was performed. Small bar indicates standard error.

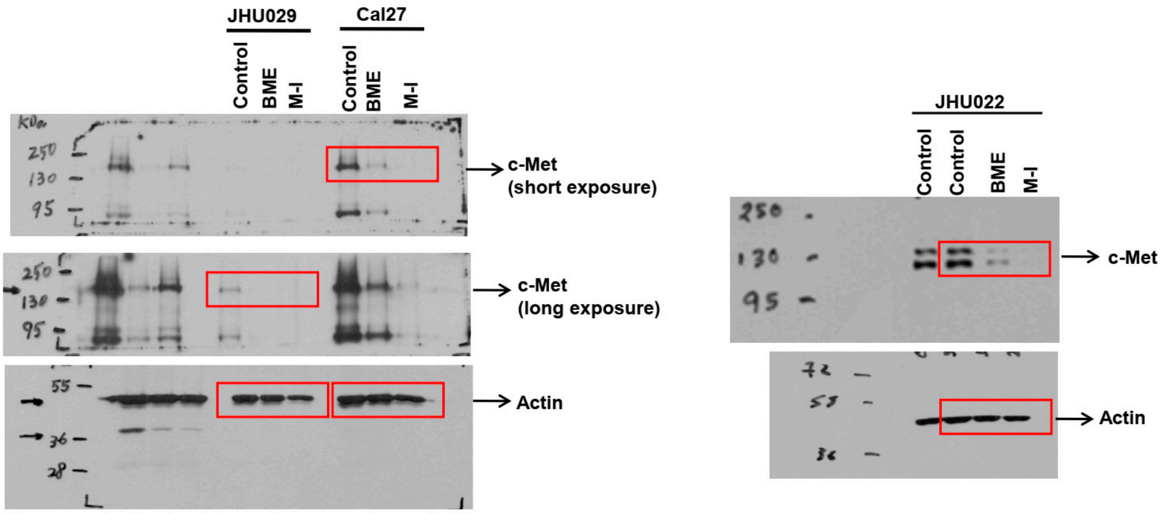

Figure S2. Whole blot for Figure 2A.

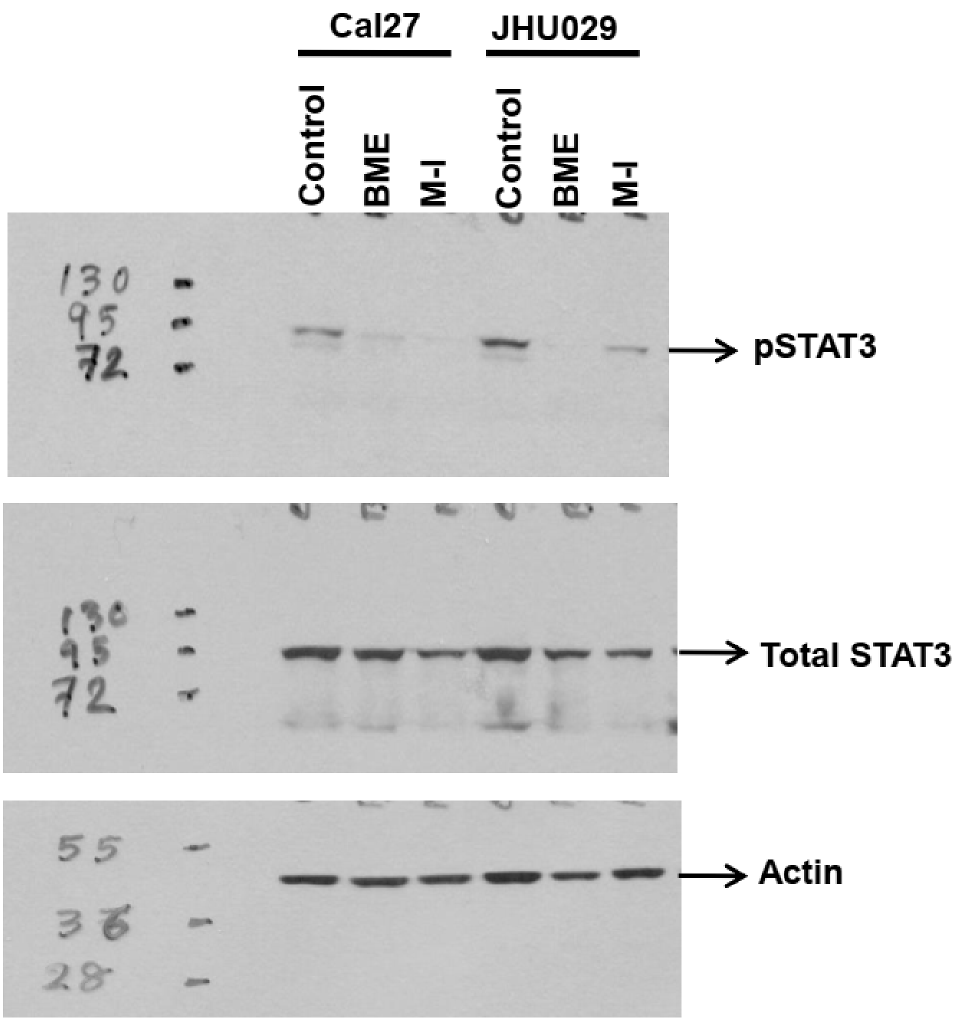

Figure S3. Whole blot for Figure 2B.

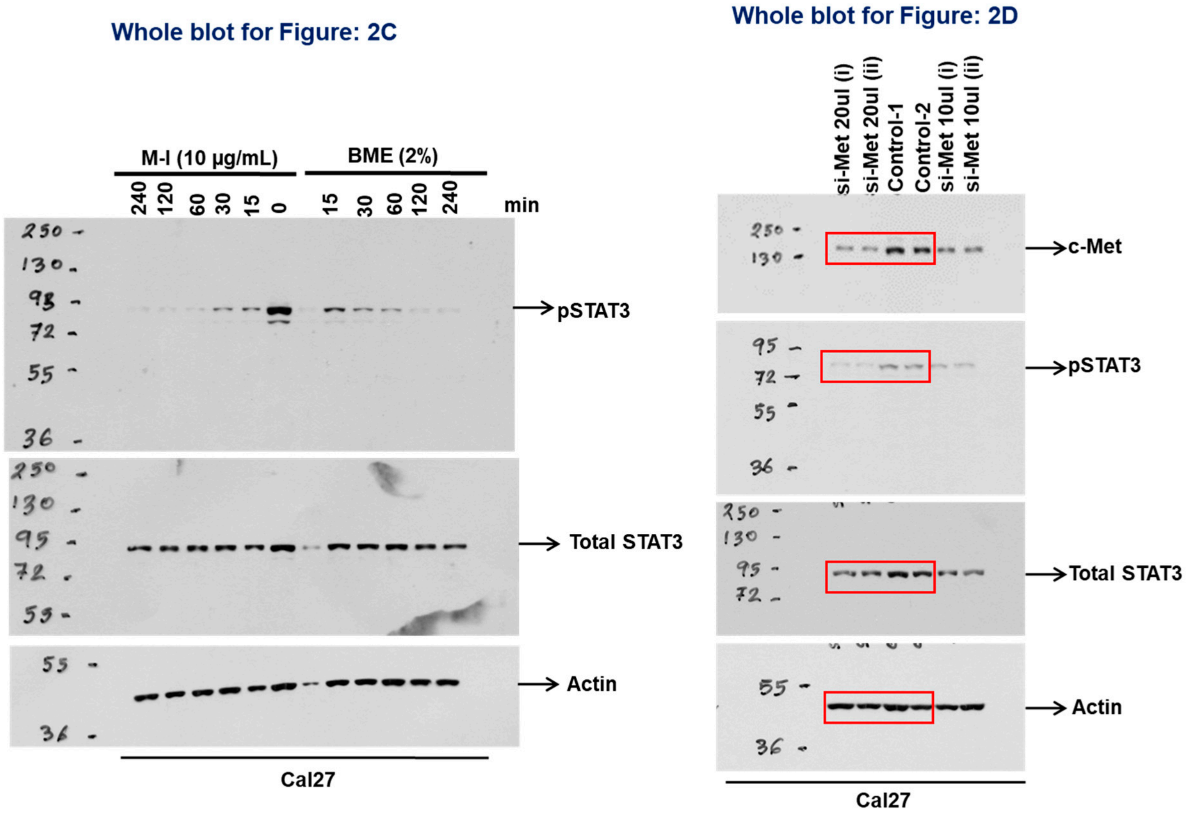

Figure S4. Whole blot for Figure 2B and 2D.

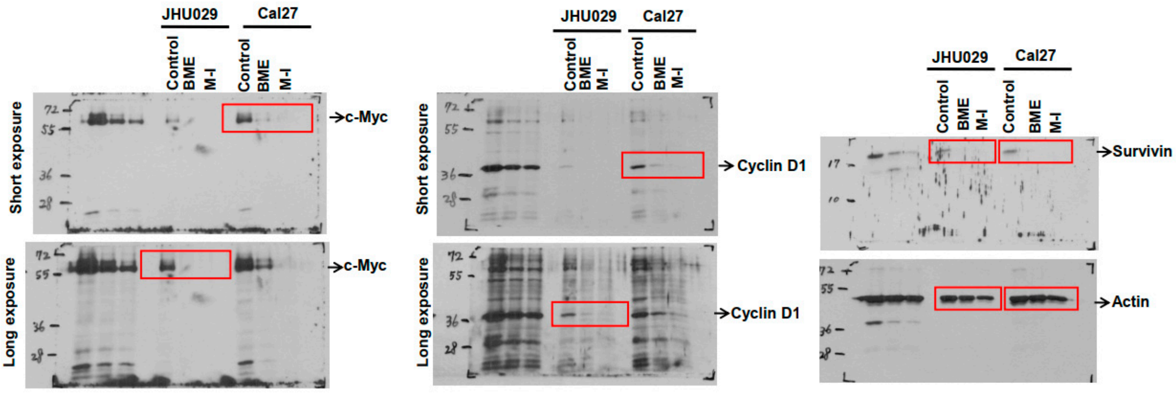

Figure S5. Whole blot for Figure 3.

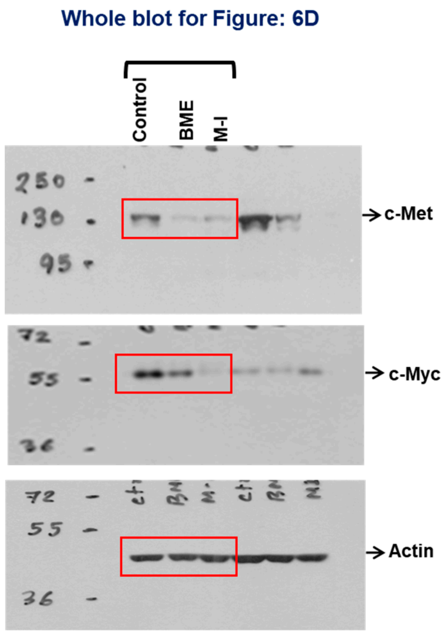

Figure S6. Whole blot for Figure 6D.

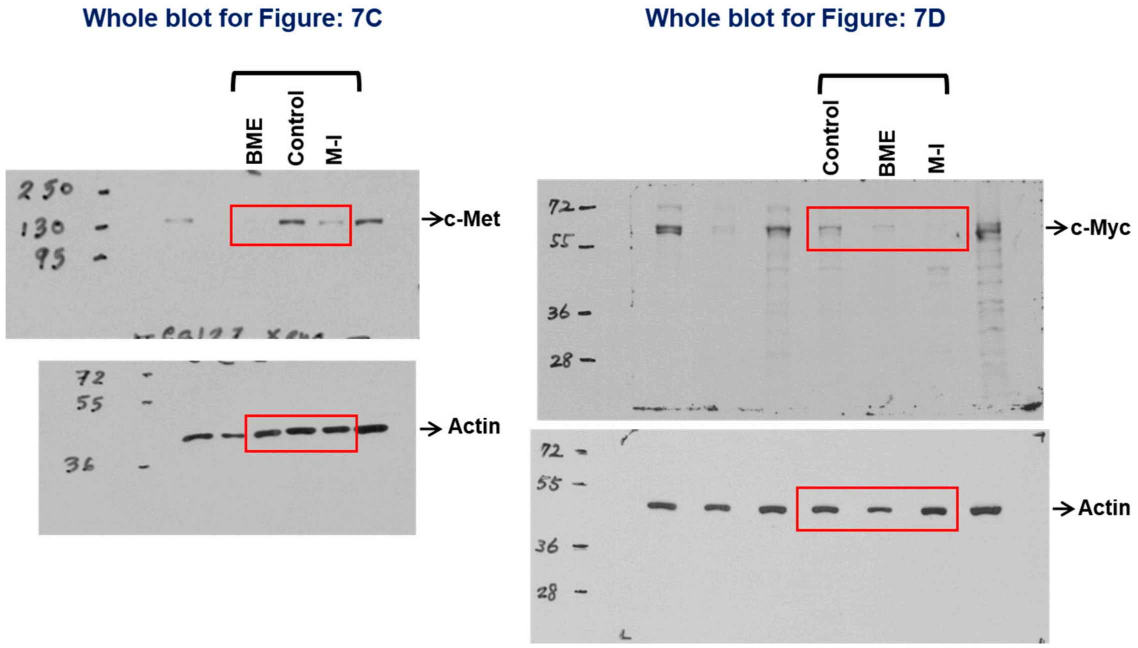

Figure S7. Whole blot for Figure 7C,D.
